# Crystal structure of 7,8,9,10-tetra­hydro­benzo[*b*]naphtho­[2,1-*d*]furan

**DOI:** 10.1107/S2056989015024512

**Published:** 2016-01-01

**Authors:** Zhongyuan Wu, Manfred T. Reetz, Klaus Harms

**Affiliations:** a Fachbereich Chemie, Philipps-Universität, Hans-Meerwein-Strasse 4, 35032 Marburg, Germany; b Max-Planck-Institut für Kohlenforschung, Kaiser-Wilhelm-Platz 1, 45470 Mülheim, Germany

**Keywords:** crystal structure, Diels–Alder reaction, Friedel–Crafts reaction, furan, tetra­hydro­benzo­naphtho­furan, C—H⋯π inter­actions

## Abstract

In the title compound, C_16_H_14_O, the cyclo­hexene ring has a half-chair conformation. The mean plane, calculated through all non-H atoms of the mol­ecule, except for the central CH_2_ atoms of the cyclo­hexene ring, which deviate by 0.340 (3) and −0.369 (3) Å from this mean plane, has an r.m.s. deviation of 0.012 Å. In the crystal, there are C—H⋯π contacts present, resulting in the formation of zigzag chains propagating along the [010] direction.

## Abstract 2

The reaction of 1-naphthol with cyclo­hexa­diene in the presence of catalytic amounts of Lewis acid, which inter­acts with 1-naphthol with release of protons, does not afford the Diels–Alder adduct but the Friedel–Crafts products followed by aromatization. The crystal structure of the final tetra­hydro­benzo­naphtho­furan product is described.

## Chemical context   

The inter­action of Lewis acids with 1-naphthol **1** can be expected to induce metal coordination at the hy­droxy function with concomitant increase in Brønsted-acidity (**2**) (Yamamoto & Futatsugi, 2005 ▸; Goering, 1995 ▸). It is conceivable that the proton, once released from this inter­mediate **2**, adds reversibly to the 4-position with formation of adduct **3**, which is the Lewis acid coordinated form of the keto-tautomer of **1**. Even if only minute amounts of **3** were to be formed, this inter­mediate should be a highly reactive dienophile in Diels–Alder reactions with such dienes as cyclo­hexa­diene **4** leading to adduct **5** (see Scheme). Such a transformation implies de-aromatization of 1-naphthol **1**.
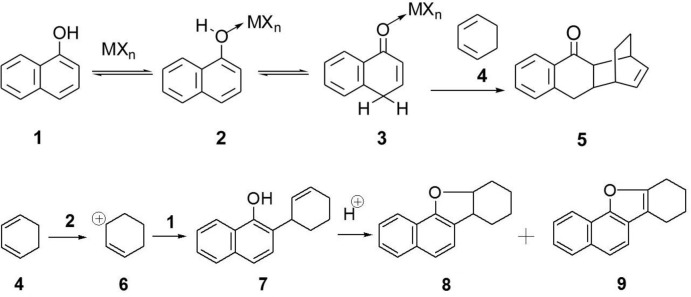


Alternatively, protonation of diene **4** leading to carbocation **6** would set the stage for Friedel–Crafts reaction with formation of the alkyl­ation product **7**, which could continue to react acid catalyzed, leading to adduct **8** and possibly to the aromatized furan product **9**. In a previous study, Novák and coworkers reported the reaction of **1** with **4** in the presence of TsOH·H_2_O in boiling toluene (26 h) or at room temperature (7 d), furan derivative **9** being formed in 58% yield, presumably *via* the inter­mediacy of **7** and **8 (**Orovecz *et al.*, 2003 ▸; Novák *et al.*, 2000 ▸).

In exploratory experiments, we tested Et_2_O·BF_3_, FeCl_3_, TiCl_4_ and ZrCl_4_ as Lewis acids in the reaction of **1** and **4** at room temperature in CH_2_Cl_2_. Essentially only products derived from formal Friedel–Crafts alkyl­ation were identified following column chromatographic separation. Small amounts of unidentified compounds which could not be separated were also formed. A general protocol is provided. If a 2.5-fold excess of cyclo­hexa­diene **4** is used in these reactions, only small amounts of Friedel–Crafts products are formed (3–4%). Rather, acid-mediated oligomerization of diene **4** occurs.

In contrast to the acidic conditions employed by Novák and coworkers, using the present protocol we isolated compound **8** and characterized it for the first time. We report herein on the crystal structure of the final product, furan **9**.

## Structural commentary   

**Figure 1:**
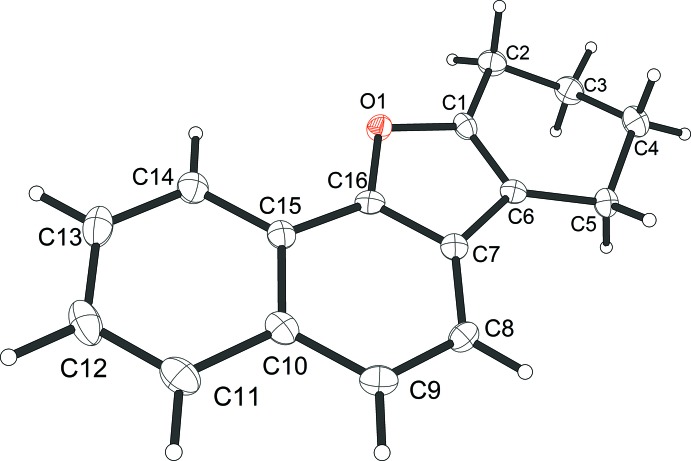
The mol­ecular structure of compound **9**, showing the atom labelling. Displacement ellipsoids are drawn at the 50% probability level.

In the title compound **9**, illustrated in Fig. 1 ▸, the cyclo­hexene ring (C1–C6) has a half-chair conformation. The mean plane, calculated through all non-hydrogen atoms of the mol­ecule (O1/C1/C2/C5–C16), except atoms C3 and C4 of the cyclo­hexene ring that deviate by 0.340 (3) and −0.369 (3) Å from this mean plane, has an r.m.s. deviation of 0.012 Å. The other C and O atoms lie in this mean plane with a maximum deviation of −0.051 (3) Å for atom C2.

## Supra­molecular features   

**Figure 2:**
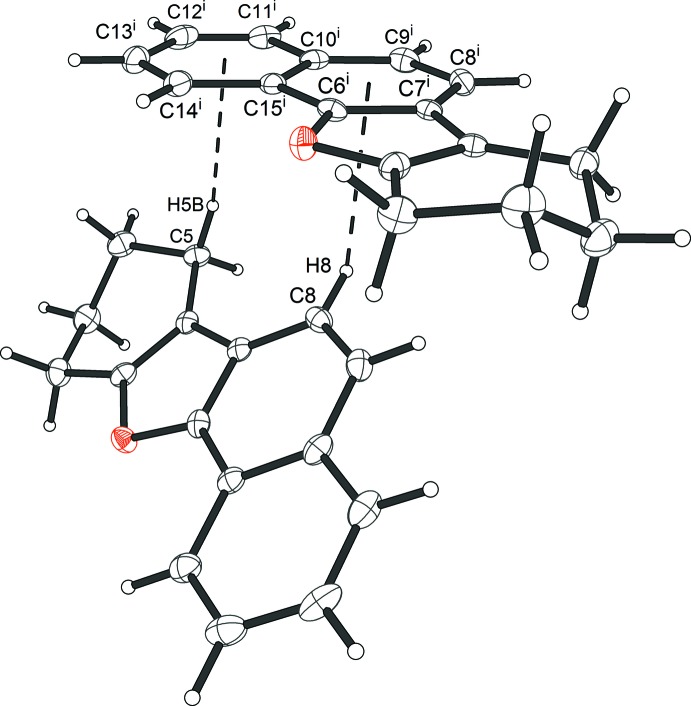
A view of the nearest C—H⋯ring centroid distances, shown as dashed lines [see Table 1 ▸; symmetry code: (i) −*x*, −*y* + 2, *z* − 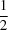].

In the crystal of **9**, there are C—H⋯π contacts present (Table 1 ▸ and Fig. 2 ▸), but no classical hydrogen bonds and no π–π inter­actions present. Inter­molecular contacts thus appear to be limited to van der Waals inter­actions. The two rather short inter­molecular C—H⋯ring centroid distances are: H5*B*⋯centroid of ring (C10–C15) = 2.69 Å, H8⋯centroid of ring (C7–C10/C15/C16) = 2.93 Å. These inter­actions result in the formation of zigzag chains propagating along the *b*-axis direction.

## Database survey   

Only one structure of a tetra­hydro­benzo­naphtho­furan (Refcode PEBDAD; Scully & Porco, 2012 ▸) is present in the current version 5.36 of the CSD (Groom & Allen, 2014 ▸), and the cyclo­hexene ring also has a half-chair conformation.

## Synthesis and crystallization   

General Procedure: To a mixture of 1-naphthol (6.48 g, 45 mmol), catalyst (2.25 mmol) in CH_2_Cl_2_ (10 ml), 1,3-cyclo­hexa­diene (0.7 ml, 22.5 mmol) in CH_2_Cl_2_ (30 ml) was added drop wise, and the resulting solution was stirred at 273 K for 5 h. After completion of the reaction (TLC) at room temperature, a cold aqueous solution of NaHCO_3_ (5%, 20 ml) was added and the mixture was extracted with CH_2_Cl_2_ (3 × 10 ml). The organic extracts were washed with water (2 ×10 mL) and dried over anhydrous Na_2_SO_4_, and concentrated in vacuum. The crude product was purified by silica column chromatography (petroleum ether) to give the desired product, which was identified by NMR spectroscopic comparison with authentic samples of **1**, **2** and by X-ray diffraction analysis (Fig. 1 ▸).

Compound **8**: ^1^H NMR (300 MHz, CDCl_3_, p.p.m.): δ 1.19–1.27 (*m*, 1H), 1.34–1.48 (*m*, 4H), 1.69–1.84 (*m*, 2H), 1.92–2.02 (*m*, 1H), 3.17–3.24 (*m*, 1H), 4.71*-*-4.77 (*m*, 1H), 7.16–7.18 (*m*, 1H), 7.24–7.32 (*m*, 3H), 7.66–7.69 (*m*, 1H), 7.87–7.90 (*m*, 1H); ^13^C NMR (300 MHz, CDCl_3_, p.p.m.): δ 20.50, 21.86, 27.64, 28.38, 41.41, 83.44, 120.16, 121.16, 121.60, 121.92, 125.13, 125.48, 126.55, 128.01, 134.11, 155.07.

High Resolution Mass Spectrum: (*M* + H^+^) calculated for C_16_H_16_O 225.1274; found (*M* + H^+^) 225.1275.

## Refinement   

Crystal data, data collection and structure refinement details are summarized in Table 2 ▸. H atoms were located in difference Fourier maps, but subsequently included in the refinement using a riding model: C–H = 0.95-0.99 Å with *U*_iso_(H) = 1.2*U*_eq_(C).

## Supplementary Material

Crystal structure: contains datablock(s) I. DOI: 10.1107/S2056989015024512/su5264sup1.cif

Structure factors: contains datablock(s) I. DOI: 10.1107/S2056989015024512/su5264Isup2.hkl

Supporting information file. DOI: 10.1107/S2056989015024512/su5264Isup3.cml

CCDC reference: 1429774

Additional supporting information:  crystallographic information; 3D view; checkCIF report

## Figures and Tables

**Table 1:** Hydrogen-bond geometry (Å, °) *Cg*3 and *Cg*4 are the centroids of rings C7–C10/C15/C16 and C10–C15, respectively.

*D*—H⋯*A*	*D*—H	H⋯*A*	*D*⋯*A*	*D*—H⋯*A*
C5—H5*B*⋯*Cg*4^i^	0.99	2.69	3.664 (3)	167
C8—H8⋯*Cg*3^i^	0.95	2.93	3.650 (3)	134

Symmetry code: (i) 
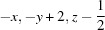
.

**Table 2:** Experimental details

Crystal data	
Chemical formula	C_16_H_14_O
*M*_r_	222.27
Crystal system, space group	Orthorhombic, *P* *n* *a*2_1_
Temperature (K)	100
*a*, *b*, *c* (Å)	13.8369 (9), 12.2202 (8), 6.8468 (4)
*V* (Å^3^)	1157.72 (13)
*Z*	4
Radiation type	Mo *K*α
μ (mm^−1^)	0.08
Crystal size (mm)	0.16 × 0.05 × 0.04

Data collection	
Diffractometer	Bruker D8 QUEST area detector
Absorption correction	Multi-scan (*SADABS*; Bruker, 2014 ▸)
*T*_min_, *T*_max_	0.94, 1.00
No. of measured, independent and observed [*I* > 2σ(*I*)] reflections	5983, 2024, 1808
*R*_int_	0.038
(sin θ/λ)_max_ (Å^−1^)	0.601

Refinement	
*R*[*F* ^2^ > 2σ(*F* ^2^)], *wR*(*F* ^2^), *S*	0.038, 0.081, 1.09
No. of reflections	2024
No. of parameters	154
No. of restraints	1
H-atom treatment	H-atom parameters constrained
Δρ_max_, Δρ_min_ (e Å^−3^)	0.16, −0.24

Computer programs: *APEX2* and *SAINT* (Bruker, 2014 ▸), *SHELXT* (Sheldrick, 2015*a* ▸), *SHELXL* (Sheldrick, 2015*b* ▸), *DIAMOND* (Brandenburg, 2006 ▸), *publCIF* (Westrip, 2010 ▸) and *PLATON* (Spek, 2009 ▸).

## supplementary crystallographic information

### Crystal data

**Table:**

C_16_H_14_O	*D*_x_ = 1.275 Mg m^−^^3^
*M**_r_* = 222.27	Mo *K*α radiation, λ = 0.71073 Å
Orthorhombic, *P**n**a*2_1_	Cell parameters from 2867 reflections
*a* = 13.8369 (9) Å	θ = 2.2–25.2°
*b* = 12.2202 (8) Å	µ = 0.08 mm^−^^1^
*c* = 6.8468 (4) Å	*T* = 100 K
*V* = 1157.72 (13) Å^3^	Prism, colourless
*Z* = 4	0.16 × 0.05 × 0.04 mm
*F*(000) = 472	

### Data collection

**Table:**

Bruker D8 QUEST area-detector diffractometer	2024 independent reflections
Radiation source: microfocus sealed X-ray tube	1808 reflections with *I* > 2σ(*I*)
Detector resolution: 7.9 pixels mm^-1^	*R*_int_ = 0.038
ω and φ scans	θ_max_ = 25.3°, θ_min_ = 2.2°
Absorption correction: multi-scan (*SADABS*; Bruker, 2014)	*h* = −16→16
*T*_min_ = 0.94, *T*_max_ = 1.00	*k* = −14→14
5983 measured reflections	*l* = −8→8

### Refinement

**Table:**

Refinement on *F*^2^	Primary atom site location: structure-invariant direct methods
Least-squares matrix: full	Secondary atom site location: difference Fourier map
*R*[*F*^2^ > 2σ(*F*^2^)] = 0.038	Hydrogen site location: inferred from neighbouring sites
*wR*(*F*^2^) = 0.081	H-atom parameters constrained
*S* = 1.09	*w* = 1/[σ^2^(*F*_o_^2^) + (0.0435*P*)^2^ + 0.0066*P*] where *P* = (*F*_o_^2^ + 2*F*_c_^2^)/3
2024 reflections	(Δ/σ)_max_ < 0.001
154 parameters	Δρ_max_ = 0.16 e Å^−^^3^
1 restraint	Δρ_min_ = −0.24 e Å^−^^3^

### Special details

**Table:**

Geometry. All e.s.d.'s (except the e.s.d. in the dihedral angle between two l.s. planes) are estimated using the full covariance matrix. The cell e.s.d.'s are taken into account individually in the estimation of e.s.d.'s in distances, angles and torsion angles; correlations between e.s.d.'s in cell parameters are only used when they are defined by crystal symmetry. An approximate (isotropic) treatment of cell e.s.d.'s is used for estimating e.s.d.'s involving l.s. planes.

### Fractional atomic coordinates and isotropic or equivalent isotropic displacement parameters (Å^2^)

**Table:**

	*x*	*y*	*z*	*U*_iso_*/*U*_eq_	
C1	0.03747 (16)	0.7566 (2)	0.3738 (4)	0.0159 (5)	
O1	0.09729 (11)	0.83253 (13)	0.4640 (2)	0.0165 (4)	
C2	0.00207 (19)	0.6599 (2)	0.4844 (4)	0.0207 (6)	
H2A	−0.0492	0.6822	0.5770	0.025*	
H2B	0.0557	0.6267	0.5596	0.025*	
C3	−0.03802 (19)	0.5773 (2)	0.3369 (4)	0.0237 (6)	
H3A	−0.0767	0.5215	0.4070	0.028*	
H3B	0.0164	0.5395	0.2716	0.028*	
C4	−0.10145 (19)	0.6329 (2)	0.1823 (4)	0.0232 (6)	
H4A	−0.1300	0.5763	0.0964	0.028*	
H4B	−0.1551	0.6719	0.2480	0.028*	
C5	−0.04412 (18)	0.7140 (2)	0.0581 (4)	0.0179 (6)	
H5A	−0.0036	0.6738	−0.0369	0.021*	
H5B	−0.0891	0.7616	−0.0156	0.021*	
C6	0.01857 (16)	0.78260 (19)	0.1870 (4)	0.0138 (5)	
C7	0.06900 (16)	0.8841 (2)	0.1498 (3)	0.0137 (5)	
C8	0.07806 (17)	0.9549 (2)	−0.0120 (4)	0.0165 (6)	
H8	0.0475	0.9381	−0.1326	0.020*	
C9	0.13190 (18)	1.0484 (2)	0.0084 (4)	0.0187 (6)	
H9	0.1371	1.0973	−0.0991	0.022*	
C10	0.18051 (17)	1.07448 (19)	0.1866 (4)	0.0171 (6)	
C11	0.23745 (18)	1.1700 (2)	0.2054 (4)	0.0215 (6)	
H11	0.2441	1.2179	0.0969	0.026*	
C12	0.28309 (18)	1.1947 (2)	0.3768 (4)	0.0243 (7)	
H12	0.3209	1.2593	0.3862	0.029*	
C13	0.27444 (18)	1.1254 (2)	0.5383 (4)	0.0234 (6)	
H13	0.3060	1.1438	0.6571	0.028*	
C14	0.22067 (17)	1.0308 (2)	0.5271 (4)	0.0191 (6)	
H14	0.2157	0.9837	0.6371	0.023*	
C15	0.17295 (17)	1.0040 (2)	0.3507 (4)	0.0151 (5)	
C16	0.11541 (17)	0.9100 (2)	0.3225 (3)	0.0141 (5)	

### Atomic displacement parameters (Å^2^)

**Table:**

	*U*^11^	*U*^22^	*U*^33^	*U*^12^	*U*^13^	*U*^23^
C1	0.0135 (11)	0.0131 (13)	0.0211 (13)	−0.0013 (10)	0.0009 (11)	−0.0021 (11)
O1	0.0202 (8)	0.0147 (9)	0.0144 (8)	−0.0019 (8)	−0.0021 (7)	0.0019 (7)
C2	0.0232 (13)	0.0180 (14)	0.0210 (14)	−0.0019 (11)	0.0019 (12)	0.0042 (12)
C3	0.0263 (14)	0.0171 (14)	0.0277 (14)	−0.0048 (12)	0.0018 (13)	0.0035 (12)
C4	0.0197 (13)	0.0213 (15)	0.0286 (15)	−0.0056 (12)	−0.0006 (12)	−0.0007 (13)
C5	0.0164 (12)	0.0169 (14)	0.0203 (13)	−0.0010 (11)	−0.0023 (11)	−0.0023 (12)
C6	0.0126 (11)	0.0128 (13)	0.0159 (12)	0.0037 (10)	0.0024 (10)	−0.0007 (11)
C7	0.0119 (12)	0.0132 (13)	0.0158 (12)	0.0041 (10)	0.0022 (11)	−0.0027 (11)
C8	0.0185 (12)	0.0172 (14)	0.0140 (12)	0.0036 (11)	−0.0010 (11)	−0.0012 (11)
C9	0.0200 (12)	0.0174 (13)	0.0186 (13)	0.0036 (11)	0.0040 (11)	0.0030 (12)
C10	0.0127 (11)	0.0153 (13)	0.0233 (14)	0.0024 (10)	0.0046 (11)	−0.0019 (12)
C11	0.0168 (13)	0.0174 (15)	0.0303 (15)	0.0005 (11)	0.0056 (13)	0.0015 (13)
C12	0.0140 (12)	0.0199 (15)	0.0391 (17)	−0.0041 (11)	0.0037 (13)	−0.0069 (14)
C13	0.0167 (12)	0.0257 (16)	0.0278 (15)	−0.0006 (12)	−0.0023 (12)	−0.0106 (14)
C14	0.0149 (12)	0.0209 (14)	0.0216 (13)	0.0012 (11)	0.0002 (11)	−0.0020 (13)
C15	0.0115 (11)	0.0159 (13)	0.0180 (12)	0.0020 (10)	0.0021 (10)	−0.0026 (11)
C16	0.0155 (12)	0.0117 (13)	0.0152 (13)	0.0035 (10)	0.0039 (11)	0.0003 (11)

### Geometric parameters (Å, º)

**Table:**

C1—C6	1.343 (3)	C7—C16	1.383 (3)
C1—O1	1.388 (3)	C7—C8	1.411 (3)
C1—C2	1.486 (3)	C8—C9	1.371 (3)
O1—C16	1.377 (3)	C8—H8	0.9500
C2—C3	1.532 (4)	C9—C10	1.429 (4)
C2—H2A	0.9900	C9—H9	0.9500
C2—H2B	0.9900	C10—C11	1.414 (4)
C3—C4	1.534 (4)	C10—C15	1.419 (3)
C3—H3A	0.9900	C11—C12	1.366 (4)
C3—H3B	0.9900	C11—H11	0.9500
C4—C5	1.528 (4)	C12—C13	1.398 (4)
C4—H4A	0.9900	C12—H12	0.9500
C4—H4B	0.9900	C13—C14	1.377 (3)
C5—C6	1.494 (3)	C13—H13	0.9500
C5—H5A	0.9900	C14—C15	1.415 (4)
C5—H5B	0.9900	C14—H14	0.9500
C6—C7	1.446 (3)	C15—C16	1.411 (3)
			
C6—C1—O1	112.4 (2)	C16—C7—C8	119.4 (2)
C6—C1—C2	127.5 (2)	C16—C7—C6	105.6 (2)
O1—C1—C2	120.1 (2)	C8—C7—C6	135.0 (2)
C16—O1—C1	104.80 (18)	C9—C8—C7	118.6 (2)
C1—C2—C3	107.9 (2)	C9—C8—H8	120.7
C1—C2—H2A	110.1	C7—C8—H8	120.7
C3—C2—H2A	110.1	C8—C9—C10	121.9 (2)
C1—C2—H2B	110.1	C8—C9—H9	119.1
C3—C2—H2B	110.1	C10—C9—H9	119.1
H2A—C2—H2B	108.4	C11—C10—C15	118.0 (2)
C2—C3—C4	111.7 (2)	C11—C10—C9	121.6 (2)
C2—C3—H3A	109.3	C15—C10—C9	120.4 (2)
C4—C3—H3A	109.3	C12—C11—C10	121.2 (3)
C2—C3—H3B	109.3	C12—C11—H11	119.4
C4—C3—H3B	109.3	C10—C11—H11	119.4
H3A—C3—H3B	107.9	C11—C12—C13	120.4 (2)
C5—C4—C3	112.0 (2)	C11—C12—H12	119.8
C5—C4—H4A	109.2	C13—C12—H12	119.8
C3—C4—H4A	109.2	C14—C13—C12	120.7 (3)
C5—C4—H4B	109.2	C14—C13—H13	119.6
C3—C4—H4B	109.2	C12—C13—H13	119.6
H4A—C4—H4B	107.9	C13—C14—C15	119.6 (2)
C6—C5—C4	109.7 (2)	C13—C14—H14	120.2
C6—C5—H5A	109.7	C15—C14—H14	120.2
C4—C5—H5A	109.7	C16—C15—C14	124.6 (2)
C6—C5—H5B	109.7	C16—C15—C10	115.3 (2)
C4—C5—H5B	109.7	C14—C15—C10	120.1 (2)
H5A—C5—H5B	108.2	O1—C16—C7	111.1 (2)
C1—C6—C7	106.1 (2)	O1—C16—C15	124.5 (2)
C1—C6—C5	122.8 (2)	C7—C16—C15	124.4 (2)
C7—C6—C5	131.1 (2)		
			
C6—C1—O1—C16	0.3 (2)	C15—C10—C11—C12	−0.7 (4)
C2—C1—O1—C16	−179.7 (2)	C9—C10—C11—C12	179.5 (2)
C6—C1—C2—C3	15.0 (3)	C10—C11—C12—C13	0.1 (4)
O1—C1—C2—C3	−165.0 (2)	C11—C12—C13—C14	0.6 (4)
C1—C2—C3—C4	−44.6 (3)	C12—C13—C14—C15	−0.6 (4)
C2—C3—C4—C5	63.3 (3)	C13—C14—C15—C16	−179.5 (2)
C3—C4—C5—C6	−45.0 (3)	C13—C14—C15—C10	−0.1 (4)
O1—C1—C6—C7	−0.5 (3)	C11—C10—C15—C16	−179.8 (2)
C2—C1—C6—C7	179.5 (2)	C9—C10—C15—C16	0.0 (3)
O1—C1—C6—C5	−179.9 (2)	C11—C10—C15—C14	0.7 (3)
C2—C1—C6—C5	0.0 (4)	C9—C10—C15—C14	−179.5 (2)
C4—C5—C6—C1	14.8 (3)	C1—O1—C16—C7	0.0 (2)
C4—C5—C6—C7	−164.5 (2)	C1—O1—C16—C15	179.4 (2)
C1—C6—C7—C16	0.4 (2)	C8—C7—C16—O1	179.21 (19)
C5—C6—C7—C16	179.8 (2)	C6—C7—C16—O1	−0.2 (3)
C1—C6—C7—C8	−178.9 (3)	C8—C7—C16—C15	−0.2 (3)
C5—C6—C7—C8	0.5 (4)	C6—C7—C16—C15	−179.6 (2)
C16—C7—C8—C9	−0.9 (3)	C14—C15—C16—O1	0.8 (4)
C6—C7—C8—C9	178.4 (2)	C10—C15—C16—O1	−178.7 (2)
C7—C8—C9—C10	1.4 (3)	C14—C15—C16—C7	−179.9 (2)
C8—C9—C10—C11	178.8 (2)	C10—C15—C16—C7	0.6 (3)
C8—C9—C10—C15	−1.0 (4)		

### Hydrogen-bond geometry (Å, º)

Cg3 and Cg4 are the centroids of rings C7–C10/C15/C16 and C10–C15, respectively.

**Table:**

*D*—H···*A*	*D*—H	H···*A*	*D*···*A*	*D*—H···*A*
C5—H5*B*···*Cg*4^i^	0.99	2.69	3.664 (3)	167
C8—H8···*Cg*3^i^	0.95	2.93	3.650 (3)	134

Symmetry code: (i) −*x*, −*y*+2, *z*−1/2.

## References

[bb1] Brandenburg, K. (2006). *DIAMOND*. Crystal Impact GbR, Bonn, Germany.

[bb2] Bruker (2014). *APEX2*, *SAINT* and *SADABS*. Bruker AXS Inc., Madison, Wisconsin, USA.

[bb3] Goering, B. K. (1995). PhD dissertation, Cornell University, Ithaca, USA.

[bb4] Groom, C. R. & Allen, F. H. (2014). *Angew. Chem. Int. Ed.* **53**, 662–671. 10.1002/anie.201306438 24382699

[bb5] Novák, L., Kovács, P., Kolonits, P., Orovecz, O., Fekete, J. & Szántay, C. (2000). *Synthesis*, **6**, 809–812.

[bb6] Orovecz, O., Kovács, P., Kolonits, P., Kaleta, Z., Párkányi, L., Szabó, É. & Novák, L. (2003). *Synthesis*, **7**, 1043–1048.

[bb7] Scully, S. S. & Porco, J. A. Jr (2012). *Org. Lett.* **14**, 2646–2649. 10.1021/ol3010563 PMC3433630 22571279

[bb8] Sheldrick, G. M. (2015*a*). *Acta Cryst.* A**71**, 3–8.

[bb9] Sheldrick, G. M. (2015*b*). *Acta Cryst.* C**71**, 3–8.

[bb10] Spek, A. L. (2009). *Acta Cryst.* D**65**, 148–155. 10.1107/S090744490804362X PMC2631630 19171970

[bb11] Westrip, S. P. (2010). *J. Appl. Cryst.* **43**, 920–925.

[bb12] Yamamoto, H. & Futatsugi, K. (2005). *Angew. Chem. Int. Ed.* **44**, 1924–1942. 10.1002/anie.200460394 15770618

